# Do interracial friendships increase the odds of having an interracial romantic relationship later in adolescence?

**DOI:** 10.1111/jora.70186

**Published:** 2026-04-25

**Authors:** Nicole S. J. Dryburgh, Brandon Cho, Naomi G. Kline, Jaana Juvonen

**Affiliations:** ^1^ Offord Centre for Child Studies McMaster University Hamilton Ontario Canada; ^2^ Department of Psychiatry and Behavioural Neurosciences McMaster University Hamilton Ontario Canada; ^3^ Department of Psychology Harvard University Cambridge Massachusetts USA; ^4^ Department of Psychology University of California, Los Angeles Los Angeles California USA

**Keywords:** adolescence, friendship, interracial relationships, romantic relationships

## Abstract

Despite growing racial and ethnic diversity in North America, interracial romantic relationships remain rare among youth. This study tested whether forming an interracial friendship by the end of middle school predicted interracial romantic relationships by the end of high school, beyond opportunities provided by school demographics. Data were from a longitudinal study of 2418 students followed from grades 8 to 12 from 26 racially diverse California public schools. Of those in relationships, 32.6% had an interracial partner. Greater availability of cross‐race peers in high school increased the odds of interracial romantic relationships, *OR* = 2.23, *p* < .001. Having at least one earlier interracial friendship 4 years earlier nearly doubled this likelihood, *OR* = 1.86, *p* < .001. Findings underscore the role of school diversity and continuity across close relationship types.

## INTRODUCTION

Marked by growing racial and ethnic diversity, the demographic landscape of youth in North America is changing (Government of Canada, 2023; US Census Bureau, 2021). Despite this increased diversity, close interracial peer relationships remain relatively rare among adolescents, even in racially diverse school contexts where opportunities for intergroup contact are high (Joyner & Kao, 2000; Moody, 2001). As such, close relationships—particularly romantic relationships—between individuals of different racial or ethnic identities are viewed as a meaningful indicator of social integration and boundary permeability between groups (Quillian & Campbell, 2003; Yancey, 2002). As romantic relationships involve deeper intimacy than most peer relationships and are salient within peer contexts (Collins et al., 2009), interracial romantic involvement offers a particularly revealing window into adolescents' willingness to cross racial boundaries in meaningful ways. Extending the developmental research on interracial friendships, the current study focuses on romantic relationships developed in high school. The goal was to examine how contextual conditions and earlier relevant relational experiences (interracial friendships) predict the likelihood of having a romantic partner of a different race by the end of high school.

Prior research has documented predictors of attitudes toward dating interracially, predominantly in college or adult samples (Herman & Campbell, 2012; Johnson & Jacobson, 2005; Thai et al., 2022), but relatively little is known about developmental precursors that shape engagement in interracial romantic relationships, especially during the period of middle adolescence when dating becomes increasingly frequent. The most obvious contextual condition that needs to be met is proximity to, or availability of, potential cross‐race or cross‐ethnic partners. Given the dominant role of schools in shaping youths' social lives, the opportunities provided by the ethnic and racial diversity of schools are particularly important contextual factors to consider (Graham et al., 2014). Most importantly for the current investigation, the role of prior relevant cross‐group relational experiences in increasing the odds of inter‐racial romantic involvement was examined. The question is whether there is developmental continuity between intimate, platonic, predominately same‐sex relationships and subsequent interracial romantic involvement across early and middle adolescence.

Before proceeding with a review of the relevant background for the current study, a brief note on terminology is needed. When referring to research on cross‐race or cross‐ethnic relational ties, the current paper at times uses ethnicity (e.g., cross‐ethnic friendships) but other times race (e.g., interracial romantic relationships), reflecting the terms used in respective studies. For the current analyses, the term interracial rather than cross‐ethnic was used to refer to friendships and romantic ties. The implication is not that race and ethnicity are synonymous, but one term is used for the sake of parsimony.

### Schools contexts and early interracial friendships

Schools represent a critical developmental context for the formation of interracial relationships when they are racially diverse (Graham et al., 2014; see Kornienko & Rivas‐Drake, 2022). Diverse contexts provide opportunities to form close, voluntary, and emotionally significant relationships across racial groups. According to intergroup contact theory (Allport et al., 1954; Pettigrew, 1998), positive contacts and close relationships between members of different social groups, in turn, reduce prejudice, promote empathy, and increase comfort with future intergroup interactions (Pettigrew & Tropp, 2006). Additionally, there is evidence, still emerging, that participation in one interracial relationship may increase openness to interracial relationships of other forms (Davies et al., 2011; Jacobson & Johnson, 2006; Thai et al., 2022).

Friendship may be a particularly powerful form of intergroup contact because these relationships are voluntary, reciprocal, and emotionally intimate (Bagwell & Bukowski, 2018; Dryburgh et al., 2022; Schwartz‐Mette et al., 2020). These relational characteristics likely make friendship especially conducive to reducing intergroup anxiety and fostering attitudinal change (Davies et al., 2011). During adolescence, friendships also serve as important contexts for skill development, providing opportunities for youth to practice conflict resolution, emotional regulation, and intimacy (Bagwell & Bukowski, 2018; Dryburgh et al., 2022). Friends also shape emerging attitudes and expectations, including those related to other‐race peers (Aboud & Sankar, 2007), thus likely increasing the likelihood of subsequently forming interracial romantic relationships. Indeed, in one other study that examined this question, the percentage of youths' cross‐race friendships across grades 7 to 12 was shown to predict interracial romantic relationships in adulthood (Kao et al., 2019). Our study extends this work by shifting the focus from the overall proportion of cross‐race friendships across adolescence to the presence of at least one interracial friendship in middle school (grade 8) and its role in predicting interracial relationships by the end of high school (grade 12). The current study proposes that forming close, interracial friendships should increase comfort with cross‐group intimacy, expand perceptions of who is considered a viable romantic partner, and normalize interracial connections within peer networks (Killen et al., 2022). In this way, early interracial friendships may scaffold a developmental progression toward interracial romantic relationships, particularly in adolescence—an age at which both peer influence and romantic exploration are salient (Benner & Wang, 2017; Collins, 2003).

### Current study

The primary aim of the present study was to shed light on the developmental continuity between earlier interracial friendship and later interracial romantic relationships, thereby extending research on intergroup contact into the domain of adolescent romantic development. In doing so, this study contributes to broader literatures on adolescent relationships by highlighting the role of early peer experiences in shaping later romantic partner selection and the developmental progression from friendship‐based intimacy to romantic involvement. By linking friendship and romantic relationship contexts across time, this study bridges domains that are often examined separately and focuses on key developmental periods when both peer relationships and romantic exploration become increasingly salient.

This study addresses gaps in the existing literature in three key ways. First, although prior research has examined predictors of interracial romantic relationships in adulthood (Kao et al., 2019), there is a dearth of longitudinal research on this topic that bridges early and middle adolescence, when youth are first exploring the formation of romantic ties. As such, for the current study, the end of middle school (grade 8) and end of high school (grade 12) were chosen as the two time points when youth have had ample opportunities to know and form close relationships with their schoolmates. Second, this study leveraged a large, racially diverse sample and a prospective design to examine whether early interracial friendship predicts later interracial romantic involvement. In addition to testing this primary hypothesis, the sample allowed for exploratory tests of moderation by racial group and gender, including their intersection, to examine whether patterns of interracial romantic involvement vary across demographic subgroups. Prior research suggests that both racial identity and gender shape patterns of interracial relationship formation. For example, gender asymmetries in interracial marriages have been documented and suggest that the social meanings and acceptability of interracial relationships may differ for men and women of the same race (Livingstone & Brown, 2017). Building on this work, the current study examined whether developmental links differed across racial groups and gender. Third, consistent with past research on cross‐ethnic romantic relationships (Clark‐Ibanez & Felmlee, 2004), analyses accounted for the opportunities provided by the school environment to form interracial relationships. Specifically, the study considers the independent effect of the high school racial composition, operationalized as the relative proportion of cross‐race (as opposed to same‐race) peers available as potential partners. Specific hypotheses were as follows:

Hypothesis 1 Greater availability of opportunities to form interracial relationships, assessed as the proportion of cross‐race schoolmates at one's high school, will predict a greater likelihood of being in an interracial romantic relationship in high school/middle adolescence.

Hypothesis 2 Over and above the availability of opportunities to form interracial relationships, having at least one interracial friendship by the end of middle school, that is, in early adolescence, will predict a greater likelihood of having an interracial romantic partner by the end of high school, that is, in late adolescence.

## MATERIALS AND METHODS

The current study leverages data from the UCLA Middle and High School Diversity project, a longitudinal study following students from 26 racially diverse (Simpson's index ranged from 0.5 to 0.77) public schools in California. The initial full sample consisted of 3148 students who participated at both grades 8 and 12. To examine the role of school‐based contextual conditions (i.e., opportunities to form interracial relationships), those who had a romantic relationship with someone outside of their school in grade 12 were excluded (*n* = 714). To further account opportunities to form interracial relationships, the analytic sample was restricted to youth who reported a racial identity that was represented by more than 5% of the sample. Youth were also included only if they reported a single racial identity (i.e., were not multiracial), due to demonstrated complexities associated with third‐party judgments of racial similarity of the close relationships of multiracial youth (Kline, 2025). After applying these criteria, the final analytic sample for the current study consisted of 2418 students.

Based on self‐reported sex (note that only two options were provided), the final sample was 51.6% female youth and 48.4% male youth. Based on self‐reported race, the sample was 11.6% Black; 22.6% East or Southeast Asian; 37.8% Latino or Mexican, and 28.0% White or Middle Eastern. The majority of the sample (63.2%) was not in a relationship in grade 12. Of those who reported being in a romantic relationship in grade 12, the majority (98.8%) reported being in a heterosexual romantic relationship, 1.2% reported a relationship with a same‐sex partner, and 0.6% reported their partner's gender as either transgender, gender fluid, gender non‐conforming, or questioning.

### Measures

#### Interracial friendship in grade 8

To measure friendships, peer nominations were utilized. Youth were asked to nominate an unlimited number of good friends from a roster of youth who were in their grade at school. Next, they were asked to report, for each nominated friend, whether that friend was the same race as the participant (0 = no, 1 = yes). These subjective reports from grade 8 were used to code whether the participant had at least one interracial friendship (1) or not (0). Subjective reports by participants were used (rather than friend‐reported ethnic identity).

#### Interracial romantic relationship in grade 12

Youth were asked developmentally sensitive questions about their romantic relationships in grade 12. Specifically, they were asked a series of questions ranging from having an exclusive romantic relationship (24.2%), a non‐exclusive romantic relationship (1.1%), casually dating (9.6%), or not involved in a romantic relationship (63.2%; note: 1.9% did not disclose).

Participants who reported being in any type of romantic relationship (*n* = 747) were then asked to indicate their partner's race or ethnicity, as well as their own race or ethnicity by selecting which of 10 options best described their identity along with an open‐response option. Responses were recoded to match the categories used in the California Department of Education (CDE) surveys, as described above, and then matched to determine whether they were in an interracial romantic relationship (1; i.e., reported the same race for themselves and their partner) or not (0).

### Additional predictors

***Availability of interracial peers*** in grade 12 was based on the school racial composition reported on the California Department of Education website and coded as the percentage of students who were of a different race than each participant's self‐report. Responses were reverse‐scored so that greater responses represented more availability, or opportunity, to form interracial relationships. Participant ***race*** was based on self‐reports at grade 12 when the assessment of interracial romantic ties was determined. In the analyses, race was dummy‐coded. Additionally, the analyses took into account ***sex*** based on self‐reported sex at grade 8 (0 = male; 1 = female).

### Procedure

This study was approved by the University of California Institutional Review Board. Students were recruited by trained research assistants visiting homerooms to distribute information letters and consent forms. Written consent from caregivers and assent from students were obtained at the onset of the middle school study and then again at the onset of the high school. In middle school, 81% of guardians provided consent and 83% of students gave assent; participation rates ranged from 74% to 94% (mean = 84%) across the 26 middle schools. Questionnaires took approximately one hour to complete, and participants were compensated with $10 in middle school and up to $20 in high school.

### Analytic plan

A logistic regression analysis (generalized estimating) was used to test whether having at least one interracial friendship in grade 8 was associated with a greater likelihood of being in an interracial romantic relationship in grade 12, while accounting for clustering of participants within schools. Specifically, at grade 8, participants were nested within 26 racially diverse California middle schools, and clustering at the school level was accounted for in analyses. School‐level identifiers were not available at the Grade 12 wave, but analyses included a participant‐level measure of interracial peer availability (i.e., the percentage of students at the participant's school who were of a different race than the participant). Control variables were included to examine the likelihood of associations holding for sex and this availability of interracial peers in high school. Odds ratios were calculated to represent the increase in likelihood of being in an interracial romantic relationship in grade 12 based on having at least one interracial friendship in grade 8. To account for potential racial differences in forming interracial romantic relationships, race was dummy‐coded and White youth were used as the comparison group. Moderation analyses were conducted to examine whether associations involving race differed by sex by including interaction terms between sex and dummy‐coded racial identity variables. Significant interactions were probed by estimating predicted probabilities of interracial romantic involvement in grade 12 across levels of the moderator and by sex, which were plotted for interpretation. Robust standard errors were estimated to ensure valid inference. All analyses were performed in SPSS version 30.

## RESULTS

Reflecting the racial diversity of the middle schools (Graham et al., 2009), 80.4% of students had at least one interracial friendship at grade 8. At grade 12, about one‐third (32.6%) of those in a relationship reported having a romantic partner who was not of the same race, whereas 67.4% of romantic relationships were same race. As such, compared to platonic close ties, romantic relationships with someone of a different race are much less common.

**FIGURE 1:**
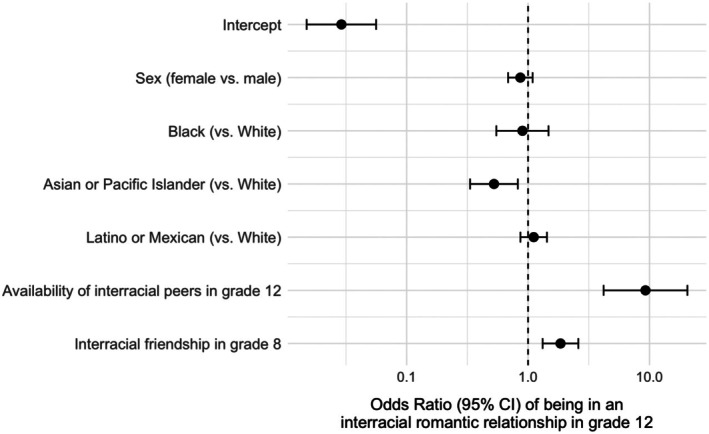
Forest plot of model odds ratios and 95% CIs.

Table 1 depicts the results of the main logistic regression analyses predicting the likelihood of an interracial romantic relationship by the end of high school. Turning first to demographic differences, no significant differences were found between female and male youth, while only one racial difference was documented: East or Southeast Asian youth had 48% lower odds of being in an interracial romantic relationship compared with White youth, *OR* = 0.52, *B* = −0.65, *p* = .005. Consistent with expectations, the high school racial composition was associated with increased odds of an interracial romantic relationship, by 9.30‐fold, *B* = 2.23, *p* < .001. Over and above the availability of interracial peers, having at least one interracial friendship in middle school significantly predicted a greater likelihood of being in an interracial romantic relationship in grade 12, *OR* = 1.86, *B* = 0.62, *p* < .001. See Figure 1 for a graphical comparison of model‐predicted odds ratios.

**TABLE 1:** Prediction of being in an interracial romantic relationship in grade 12.

	Outcome: interracial romantic relationship in grade 12			
	B	SE	Wald Chi^2^	Odds ratio
Intercept	−3.53	0.33	113.80	0.03***
Sex (female, male)	−0.15	0.12	1.51	0.86
Black	−0.11	0.25	0.18	0.90
Asian	−0.65	0.23	7.86	0.52**
Latino or Mexican	0.11	0.13	0.70	1.12
Availability of interracial peers in grade 12	2.23	0.41	30.15	9.30***
Interracial friendship in grade 8	0.62	0.17	12.69	1.86***

*Note*: To account for potential school‐level differences, analyses account for clustering of participants within schools in grade 8. The reference category for racial group was White.

** *p* < .01.

*** *p* < .001.

**FIGURE 2:**
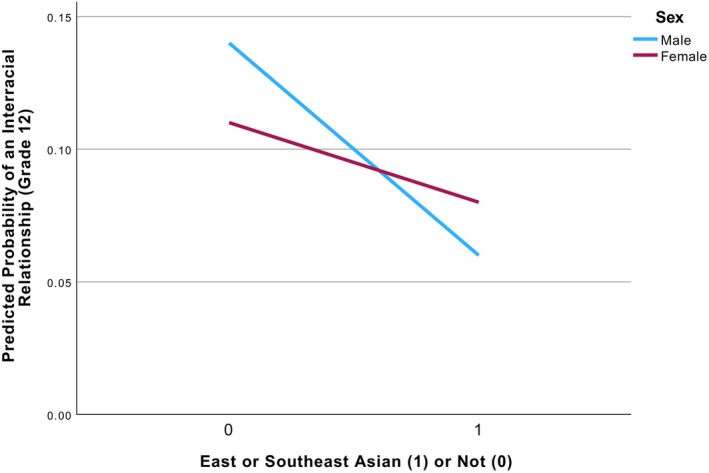
Moderation between sex and East or Southeast Asian Identity. Predicted probability of reporting an interracial romantic relationship in Grade 12 as a function of sex and East or Southeast Asian identity. Estimates are derived from a logistic regression model controlling for racial identity, having an interracial friendship in grade 8, and school racial composition in grade 12.

There was a significant interaction between sex and East or Southeast Asian identity, *Wald χ* ^*2*^(1) = 4.68, *p* = .03, indicating that the association between having an East or Southeast Asian identity and the likelihood of an interracial romantic relationship in grade 12 differed between male and female youth. As displayed in Figure 2, among non‐East or Southeast Asian youth, male youth had a slightly higher predicted probability of reporting an interracial romantic relationship than did female youth (.14 vs. .11). In contrast, among East or Southeast Asian youth, female youth had a slightly higher predicted probability of an interracial romantic relationship in grade 12 than did male youth (.08 vs. .06).

## DISCUSSION

Interracial marriages were not allowed in the United States until 1967 with the landmark decision of *Loving v. Virginia*, and remain rare, with rates of interracial marriages at only 17% in 2017 (Livingstone & Brown, 2017). This number marks a historical increase in interracial romantic ties among adults—that is, from the 1970 rate of 3%—and there has also been a steady increase in the racial and cultural diversity of North American youth (US Census Bureau, 2021). Yet, fairly little is known about interracial romantic relationships among adolescents. Extending findings documenting that interracial relationships in adulthood are more likely among those who have had a best friend or a greater proportion of peers of another race (Kao et al., 2019), the current findings underscore the critical role of school diversity and earlier interracial experiences in increasing the chances of youth having an interracial romantic relationship by the end of their compulsory education. The current findings also suggest that the threshold for forming a close interracial tie in school is lower for platonic relationships earlier in development than for forming a subsequent intimate relationship later in adolescence. Finally, these findings offer rare longitudinal evidence of experiences across multiple relationship contexts during adolescence and underscore the influential role of friendship in shaping youth development.

Contextual conditions of school racial composition are critical in providing opportunities for cross‐group interactions and relationships. Consistent with prior research demonstrating that greater school diversity is associated with greater diversity of friends (Chan & Benner, 2025), the current findings document that over 80% of youth included in the current longitudinal analyses had at least one interracial friendship. The markedly lower prevalence of interracial romantic relationships (about 30% of those in a romantic relationship) relative to friendships suggests that the threshold for crossing racial boundaries in romantic contexts is considerably higher, likely reflecting the influence of stronger social and cultural norms around romantic connections, and perhaps competing factors such as parental socialization and expectations. Moreover, the developmental context matters: many adolescents were not in any romantic relationship at all, making dating outside of one's racial group an especially unconventional choice that departs both from the norm of not dating and from prevailing racial boundaries. Thus, while the daily contact afforded by diverse school settings may set the stage for interracial relationships to occur, additional barriers—such as social norms or family influences – appear to constrain whether adolescents ultimately form these interracial romantic ties.

Importantly, beyond the availability of cross‐race peers, having at least one close interracial friendship in early adolescence nearly doubled the likelihood of forming an interracial romantic relationship in later adolescence. This finding highlights that peer contact matters not only in terms of exposure but also in terms of intimacy, closeness, and trust built through early cross‐race, close relationships. These findings contribute to a growing body of research highlighting the importance of peer relationships, specifically friendships, as foundational contexts for the subsequent formation of intimate and interracial bonds (Juvonen et al., 2018; Kao et al., 2019; Pettigrew & Tropp, 2006). Whereas prior studies have primarily focused on the benefits of interracial friendships for improving intergroup attitudes, our findings extend this work by demonstrating that these early relationships also lay the groundwork for deeper interracial intimacy, represented by the formation of interracial romantic partnerships. By doing so, this study provides evidence that early interracial friendships may promote comfort with interracial closeness, reshape perceptions of who is considered a viable romantic partner, and increase openness to forming emotionally significant ties across racial lines. As such, this study adds to the evidence consistent with cascade models of adolescent social development (see Collins, 2003; Masten & Cicchetti, 2010), which emphasize that early peer experiences scaffold later relational competencies and expectations. Furthermore, these findings build upon intergroup contact theory (Allport et al., 1954; Pettigrew, 1998) to demonstrate that, beyond just exposure to members of different social groups, the development of just one close, intimate interracial relationship—in particular, a friendship—can increase the likelihood of forming subsequent close interracial relationships. Interracial friendships may serve as early opportunities for adolescents to develop emotional intimacy, trust, and self‐disclosure with others from different backgrounds in ways that then help lower the threshold for exploring romantic and sexual interest over and beyond one's own racial group.

Importantly, these data still indicate a tendency toward homophily: of the youth in a romantic relationship in grade 12, 32.6% reported being in an interracial relationship, whereas 67.4% reported being in a relationship with someone of the same race. This finding mirrors work demonstrating the strong tendency of adolescents to form friendships with same‐race peers (Kogachi & Graham, 2021). That having an early interracial friendship predicted later interracial romantic relationships indicates that these early relationships are providing experiences that motivate youth to transcend the strong tendency and external pressures to conform to the normative expectation of forming relationships with same‐race peers. The proportion of youth with at least one interracial friendship in grade 8 was higher, with 80.4% reporting that at least one of their nominated friends was of a different race, reflecting the large, racially diverse, longitudinal sample employed here, and also potential progress over time toward more close, early interracial connections. This work underscores the foundational role of friendships in shaping patterns of intimacy, inclusion, and commitment, expanding on topics that are rarely studied in developmental psychology over and beyond the role of attachment styles or across child–parent and later romantic relationships. The findings add to the literature on the central role of friendship for youth development and adjustment (Bagwell & Bukowski, 2018; Dryburgh et al., 2022; Schwartz‐Mette et al., 2020), and reinforce the idea that opportunity alone is insufficient; voluntary, meaningful engagement across racial groups is key to fostering long‐term inclusion.

Importantly, the observed associations held even after accounting for structural opportunity to form interracial relationships and across different racial identities and sex. Notably, East or Southeast Asian youth had 48% lower odds of being in an inter‐racial romantic relationship than White youth, even after controlling for sex and the availability of interracial peers. Further, exploratory analyses indicated a significant interaction between sex and East or Southeast Asian identity. Among youth who did not identify as East or Southeast Asian, male youth showed a slightly higher predicted probability of reporting an interracial romantic relationship than female youth; in contrast, among East or Southeast Asian youth, female youth showed a somewhat higher predicted probability than male youth. Although modest in magnitude, this pattern may reflect broader gendered dynamics in interracial partner selection. For example, research with adults demonstrates similar asymmetries in interracial marriage patterns, with marriages between Asian women and White men occurring substantially more frequently than marriages between Asian men and White women (Livingstone & Brown, 2017). Scholars have argued that such patterns may reflect racialized gender stereotypes and broader social hierarchies shaping partner preferences and opportunities across racial groups (Auelua‐Toomey & Roberts, 2024). Though exploratory in nature, the current findings raise the possibility that gendered patterns in the transition from interracial friendship to later romantic relationships may begin to emerge even earlier in development.

### Limitations and future directions

This study has several strengths, including the leveraging of longitudinal data across early to later adolescence, in racially diverse schools, and with a large sample that allowed for a sufficient examination of youths' formation of romantic relationships. Despite these strengths, there are limitations. Most notably, biracial and multiracial youth were not included in the analyses. This limitation is similar to much other research on youth relationships (Nishina & Witkow, 2020, 2021), and one that future research is needed to address. In the current study, this exclusion was due to a lack of details and considerations for what is considered a biracial or interracial relationship for these youth (Kline & Juvonen, 2023). Although a youth‐centered approach was used to ask youth whether their friendships were interracial, future studies should apply this method to the assessment of later romantic relationships as well. This direction needs to be urgently addressed given that research indicates that biracial youth have specific interpersonal experiences with peers such as receiving more acceptance and rejection nominations from out‐group peers than do White students (Kline & Juvonen, 2023). This inclusion will be particularly important given the rapid growth of the multiracial youth population and the urgent need for research that reflects their unique and often overlooked experiences.

Additionally, although school‐level racial composition data were obtained from the California Department of Education and used to construct a participant‐level measure of interracial peer availability at grade 12, school identifiers were not retained in the analytic dataset. As a result, it was not possible to report the number or racial composition of the distinct high schools attended by participants at grade 12. Future study may benefit from examining potential school‐level differences in associations in addition to modeling individual‐level opportunities for interracial contact.

Finally, although the current study included relationships between all genders and did not restrict analyses to only same‐sex friendship, an important limitation was that gender identity was not assessed. This methodological constraint limited the ability to distinguish between sex and gender or to examine whether results held for youth of differing gender identities. This omission also constrains our ability to consider how intersecting social identities may jointly shape relationship formation. For example, recent research indicates that intersections of racial and sexual identities may be meaningfully associated with patterns of dating in adolescence (e.g., Cutri et al., 2025). Future research would therefore benefit from assessing gender using more inclusive measures, particularly given evidence that nearly 10% of youth report a gender‐diverse identity (Kidd et al., 2021). Youth who hold multiple marginalized identities may also experience a more limited choice of “ingroup;” as such, examining when their relationship formation crosses various social boundaries can inform interventions that seek to promote these developmentally significant relationships. Overall, the findings of this study underscore interracial friendship as an early opportunity for youth to challenge persistent social boundaries; now, future research is needed to more precisely examine *for whom* these patterns persist.

## CONCLUSION

This study provides critical evidence that having early interracial friendships can serve as a foundation to forming later interracial romantic relationships, thus offering one developmental pathway through which peer experiences with others of different identities shape long‐term patterns of social integration, inclusion, and intimacy. By highlighting the role of friendships in adolescence as precursors to deeper, more salient forms of interracial connection, our findings underscore the importance of supporting close, interracial friendship as a foundation for social integration in an increasingly diverse society.

## AUTHOR CONTRIBUTIONS

**Nicole S. J. Dryburgh:** Conceptualization; writing – original draft; formal analysis; writing – review and editing. **Jaana Juvonen:** Investigation; methodology; resources; funding acquisition; writing – review and editing; project administration; supervision. **Brandon Cho:** Conceptualization; writing – review and editing; formal analysis. **Naomi G. Kline:** Writing – review and editing.

## FUNDING INFORMATION

This research was supported by grants from the National Institutes of Health (Grant 1R01HD059882‐01A2) and the National Science Foundation (0921306). The funding sources had no role in the study design, analysis of data, interpretation of results, or writing of the report.

## CONFLICT OF INTEREST STATEMENT

There are no conflicts of interest to declare.

## ETHICS STATEMENT

This study received approval from the University of California, Los Angeles Institutional Review Board (protocol numbers 11‐002066 and 12‐001106, 6/5/2020). Informed consent was obtained from all individual participants included in the study.

## PATIENT CONSENT STATEMENT

Informed consent for adolescent participants was obtained from an appropriate designee in accordance with the ethics approval guidelines.

## Data Availability

The data, analytic code, and materials necessary to reproduce the analyses presented here are not publicly accessible but are available upon reasonable request.
